# Co-inoculation of biochar and arbuscular mycorrhizae for growth promotion and nutrient fortification in soybean under drought conditions

**DOI:** 10.3389/fpls.2022.947547

**Published:** 2022-07-22

**Authors:** Dilfuza Jabborova, Kannepalli Annapurna, A. Azimov, Swati Tyagi, Kedharnath Reddy Pengani, Prakriti Sharma, K. V. Vikram, Peter Poczai, Omaima Nasif, Mohammad Javed Ansari, R. Z. Sayyed

**Affiliations:** ^1^Institute of Genetics and Plant Experimental Biology, Uzbekistan Academy of Sciences, Tashkent, Uzbekistan; ^2^Division of Microbiology, ICAR-Indian Agricultural Research Institute, New Delhi, India; ^3^Finnish Museum of Natural History, University of Helsinki, Helsinki, Finland; ^4^Department of Physiology, College of Medicine and King Khalid University Hospital, King Saud University, Riyadh, Saudi Arabia; ^5^Department of Botany, Hindu College, Moradabad, Mahatma Jyotiba Phule Rohilkhand University, Bareilly, India; ^6^Department of Microbiology, PSGVP Mandal’s S. I. Patil Arts, G. B. Patel Science and S. T. K. V. Sangh Commerce College, Shahada, India

**Keywords:** AMF, biochar, drought stress, fluorescein diacetate, phosphomonoesterase, plant growth, soybean

## Abstract

Drought is significant abiotic stress that affects the development and yield of many crops. The present study is to investigate the effect of arbuscular mycorrhizal fungi (AMF) and biochar on root morphological traits, growth, and physiological traits in soybean under water stress. Impact of AMF and biochar on development and root morphological traits in soybean and AMF spores number and the soil enzymes’ activities were studied under drought conditions. After 40 days, plant growth parameters were measured. Drought stress negatively affected soybean growth, root parameters, physiological traits, microbial biomass, and soil enzyme activities. Biochar and AMF individually increase significantly plant growth (plant height, root dry weight, and nodule number), root parameters such as root diameter, root surface area, total root length, root volume, and projected area, total chlorophyll content, and nitrogen content in soybean over to control in water stress. In drought conditions, dual applications of AMF and biochar significantly enhanced shoot and root growth parameters, total chlorophyll, and nitrogen contents in soybean than control. Combined with biochar and AMF positively affects AMF spores number, microbial biomass, and soil enzyme activities in water stress conditions. In drought stress, dual applications of biochar and AMF increase microbial biomass by 28.3%, AMF spores number by 52.0%, alkaline phosphomonoesterase by 45.9%, dehydrogenase by 46.5%, and fluorescein diacetate by 52.2%, activities. The combined application of biochar and AMF enhance growth, root parameters in soybean and soil enzyme activities, and water stress tolerance. Dual applications with biochar and AMF benefit soybean cultivation under water stress conditions.

## Introduction

Soybean is an important food legume crop globally. Seeds of soybean contain a higher content of protein (35–40%), an essential source of oil (18–22%), carbohydrate (34%), macronutrients [phosphorus (P), nitrogen (N), potassium (K)] and micronutrients such as iron (Fe), zinc (Zn), and manganese (Mn) (de Vargas et al., 2018). Soybean contains essential amino acids [histidine, lysine, threonine, phenylalanine, cysteine, glycine, ornithine, proline, and serine (Tessari et al., 2016)]. Soybean is known to stimulate soil fertility by symbiotic biological nitrogen fixation (Ciampitti and Salvagiotti, 2018).

Drought can negatively affect soybean growth, development, yield, and nitrogen fixation (Basal and Szabo, 2020; Kannepalli et al., 2021). Water stress reduces the germination of the plant (Ilyas et al., 2020; Khan et al., 2020), growth of the plant (Sheteiwy et al., 2021), and yield (Li et al., 2018; Kour et al., 2019) in legumes. Several investigated that drought stress decreases the number of nodules per plant, the number of pods in legumes (Yücel et al., 2010; Shamsizadeh et al., 2014; Ilyas et al., 2020), and decreases root length, root volume, and yield of grain (Habibzadeh et al., 2014).

Drought negatively affects plant nutrients (Tuteja and Gill, 2013) and physiological properties such as photosynthesis (Asha et al., 2021), relative water content (Sánchez-Blanco et al., 2004), and photosynthetic rate (Hashem et al., 2019) in many plants. The P content of leaves in mungbean (*Vigna radiata* L.) decreased by water stress (Habibzadeh et al., 2014). Hashem et al. (2019) investigated water stress reduced P uptake and total N in chickpea (*Cicer arietinum* L.). Drought decreases total chlorophyll contents in chickpea varieties (Khadraji and Ghoulam, 2017). Islam et al. (2011) investigated, that drought tress decreased the transpiration rate and stomatal conductance in cowpea *Vigna unguiculata*. Drought strongly affects urease, β-glycosidase, and phosphatase of soil and soil microbial activities (Sardans and Peñuelas, 2005; Nguyen et al., 2018).

Biochar contains organic matter, nutrients, and biologically important compounds (Dume et al., 2016). Numerous studies on biochar applications enhance bioenergy production, carbon sequestration, and soil fertility (Ok et al., 2015). Biochar improved soil structure and water holding capacity in water stress (Bamminger et al., 2016). It positively impacted on plant growth and development (Sun et al., 2020; Mannan et al., 2021), nodulation (Sun et al., 2020), and yield (Liu et al., 2017) in legume crops under normal conditions and under drought stress. Biochar application increased N fixation and total biomass in *Trifolium pratense* L. (Mia et al., 2014). Numerous studies have informed biochar improved plant nutrients (Ahmed et al., 2021) and physiological traits under unstressed and water stress in many crops (Jabborova et al., 2021a,b,c; Christou et al., 2022). Mannan et al. (2021) investigated the application of biochar-enhanced soil nutrients may have enhanced soybean drought tolerance. In addition, biochar enhanced soil urease, catalase, and urease activities (Chen et al., 2020).

Arbuscular mycorrhizal fungi (AMF) are the most useful soil microbes and symbiosis relationship with roots of plant (Brundrett and Tedersoo, 2018; Jabborova et al., 2021d,f). In drought stress, AMF helps increase growth, root parameters, nodulation, and yield in legume crops (Hao et al., 2019; Haghighi and Saharkhiz, 2022). Ahmed et al. (2000) reported that AMF increased pods number and nodule number in faba bean (*Vicia faba* L.). AMF improves physiological properties (Hashem et al., 2019) and mineral nutrients, especially P and N. They help tolerate drought stress in legumes (Hashem et al., 2019). Numerous studies have informed that AMF improves host plants’ development, water uptake, and nutrient in water stress (Baum et al., 2015; Zhao et al., 2015). The AMF promoted stomatal conductance and relative water content in host plants under drought stress (Augé et al., 2015). Dual application with biochar and AMF inoculation improves the photosynthetic rate, and plant growth in chickpea under water stress (Hashem et al., 2019). This study was investigated to evaluate the impact of AMF and biochar on soybean growth, root parameters, chlorophyll content, and soil enzymatic activities in drought conditions.

## Materials and methods

### Arbuscular mycorrhizal fungi, biochar, soil, and seed

The soil was collected from the farms of the Indian Agricultural Research Institute. The studied soil had the following agrochemical properties: pH 8.0, EC 0.45 ds/m, SOC 0.41%, nitrogen 167 kg/ha, P 40.3 kg/ha, P 788 kg/ha. Biochar used in the experiment was produced at 400–500°C from woody biomass. Soybean seed was procured from the “Division of Vegetable Science” and AMF from Microbiology, IARI, India.

### Experimental design

The effect of AMF and biochar on the growth and root morphological traits in soybean was investigated in a net house at the Division of Microbiology, IARI. Four treatments as control, AMF alone, biochar alone, and combined with AMF and biochar were used for the experiment. Mixing the soil with 1.0% biochar used a pot experiment. AMF biofertilizer consists of 100 spores/g and 1,200 IP/g. Seed of soybean was cultivated into plastic pots, including soil (5.0 kg). Within 40 days of plant growth in drought conditions (50% of the field capacity) were maintained. After 40 days, plant height, nodule number, dry shoot weight, and dry root weight were measured. All the experiments were performed in triplicates.

### Root parameters measurement

Soybean root morphological traits such as total root length, projected area, root surface area, root volume, and root diameter were evaluated. All roots were spread out and detected using a scanning system (Expression 4990, Epson, CA, United States) with a blue board as a background.

### Chlorophyll, N, and C contents of leaves measurement

Chlorophyll content were analyzed (SPAD-502 meter) using the leaves of soybean. Elemental Analyzer (CHNS) Eurovictor determined C and N.

### Determination of arbuscular mycorrhizal fungi spores number and microbial biomass in soil

The AMF spores number was counted using a stereomicroscope by Dare et al. (2013). The biomass C was analyzed on those described by Vance et al. (1987). The absorbance of the resulting supernatant liquid was measured at 280 nm.

### Determination of enzymes activities in soil

Alkaline phosphatase enzyme activities were analyzed according to the method of Tabatabai and Bremner (1969). The fluorescein diacetate (FDA) hydrolytic activity was determined as per the Green et al. (2006) method. The dehydrogenase activity (DHA) was analyzed by Casida et al. (1964). The experimental data were analyzed with the StatView Software using ANOVA. The magnitude of the *F* value determined the significance of the effect of treatment (*P* < 0.05 < 0.001).

## Results

In water stress, biochar alone significantly greater the height of the plant by 28.9%, the dry weight of roots by 51.5%, and a number of nodules (per plant) by 34.9% than the control (Table 1). The AMF alone significantly enhances the plant’s height by 20.0%, the dry weight of roots by 53.8%, and the number of nodules (per plant) by 69.2% over the control under water stress. However, the dual application of AMF and biochar was applied, the plant height by 31.1%, the dry weight of root by 69.2%, and the number of nodules per plant by 63.9% over control under drought conditions.

**TABLE 1:** The plant height, shoot dry weight, root dry weight, and nodule number in soybean as affected by biochar and AMF under drought condition.

Treatments	Plant height (cm)	Shoot dry weight (g)	Root dry weight (g)	Nodule number
Control	30.00 ± 0.57	1.56 ± 0.01	0.13 ± 0.01	16.67 ± 1.52
Biochar	38.67 ± 0.58*	1.62 ± 0.01	0.21 ± 0.01*	24.00 ± 1.00*
AMF	36.00 ± 1.00*	1.59 ± 0.01	0.20 ± 0.01*	21.67 ± 1.53*
Biochar + AMF	39.33 ± 0.18*	1.70 ± 0.01	0.22 ± 0.02*	27.33 ± 0.58*

Data are means of three replicates (n = 3). Asterisk differed significantly at *P < 0.05, **P < 0.01, ***P < 0.001.

The biochar exhibited a significant positive impact on the root morphological traits of soybean (Table 2). Over the control, root surface area and total root length were suddenly promoted by biochar alone, which was significantly enhanced by 56.8% and 54.9% under drought stress. Biochar alone substantially increases the projected area by 26.2%, root volume by 35.2%, and the root diameter by 45.8% more than the control. Over to the control, total root length and, surface area were enhanced due to the application of AMF alone by 27.4 and 32.5% in drought conditions. In drought stress, AMF alone visibly increased projected area by 18.5% and root diameter by 22.9%. However, AMF and biochar significantly developed root morphological parameters under drought conditions. The maximum values of total root length were observed in dual applications with AMF and biochar in water stress. Combined biochar application with AMF treatment significantly increases the root volume by 85.2%, root diameter by 72.9%, root surface area by 66.7%, and total root length by 61.9% more than the control. While in water stress conditions, this treatment discernible improved the projected area by 35.4% over the control.

**TABLE 2:** Root morphological traits in soybean as affected by biochar and AMF under drought condition.

Treatments	Total root length (cm)	Projected area (cm^2^)	Root surface area (cm^2^)	Root volume (cm^3^)	Root diameter (mm)
Control	109.54 ± 12.72	14.57 ± 0.96	5.66 ± 0.29	0.88 ± 0.03	0.48 ± 0.01
Biochar	171.76 ± 1.53*	18.40 ± 0.5*	8.77 ± 0.94*	1.19 ± 0.02**	0.70 ± 0.01*
AMF	139.57 ± 13.77*	17.27 ± 0.11*	7.50 ± 0.08*	0.96 ± 0.01*	0.59 ± 0.06*
Biochar + AMF	177.44 ± 5.75**	19.74 ± 0.99**	9.44 ± 0.81**	1.63 ± 0.07**	0.83 ± 0.03**

Data are means of three replicates (n = 3). Asterisk differed significantly at *P < 0.05, **P < 0.01, ***P < 0.001.

Generally, water stress decreased the N and C content of leaves in soybean (Table 3). Biochar treatment increases the N content by 15.7% and the C content by 52.8% of leaf under water stress. Over the control under drought stress, the application of AMF alone promoted the N content by 10.0% and the C content by 48.4%. The C and N content reached a maximum at dual applications with AMF and biochar than to all treatments. In water stress, dual AMF and biochar treatment suggestively promoted the C content by 59.3% and N content by 16.5% more than the control.

**TABLE 3:** The nitrogen and carbon content of leaf in soybean as affected by biochar and AMF under drought condition.

Treatments	N (%)	C (%)
Control	2.459 ± 0.09	28.789 ± 2.01
Biochar	2.846 ± 0.05*	43.998 ± 1.22*
AMF	2.705 ± 0.02*	42.749 ± 5.36*
Biochar + AMF	2.867 ± 0.03*	45.889 ± 1.34*

Data are means of three replicates (n = 3). Asterisk differed significantly at *P < 0.05, **P < 0.01, ***P < 0.001.

**FIGURE 1:**
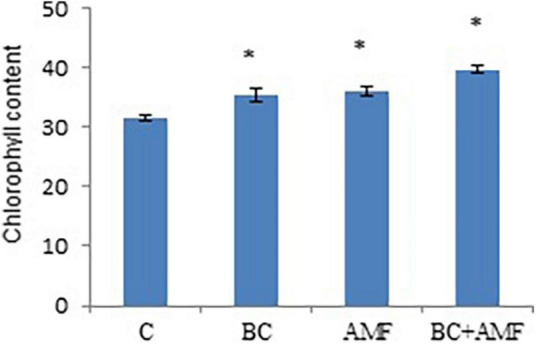
The chlorophyll content of leaves in soybean as affected by biochar and AMF under drought conditions. C, control; BC, biochar; AMF, arbuscular mycorrhizal fungi; BC + AMF, biochar + arbuscular mycorrhizal fungi. *P = 0.01.

Water stress declines total chlorophyll content in soybean. In water stress, biochar alone significantly enhances the chlorophyll content in soybean by 12.9%. The application of AMF alone increased chlorophyll content by 14.9% over the control. Highest values of the chlorophyll content were observed at the dual application of AMF, and biochar increased significantly by 16.5% (Figure 1).

**FIGURE 2:**
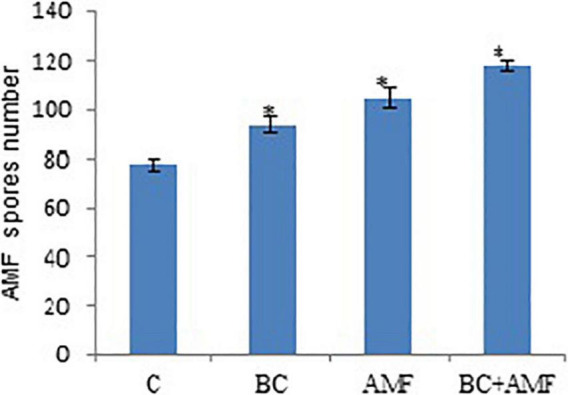
The AMF spore numbers in soil affected by biochar and AMF under drought conditions. C, control; BC, biochar; AMF, arbuscular mycorrhizal fungi; BC + AMF, biochar + arbuscular mycorrhizal fungi. *P = 0.01.

The number of spores in AMF, increases from 34.7% in drought stress. The application of applying biochar treatment stimulated the number of spores significantly in AMF under water stress conditions. The biochar alone increases the number of spores of AMF considerably by 20.6%, respectively. The dual application of AMF and biochar was more beneficial in enhancing the number of AMF spores in the soil. However, dual AMF and biochar meaningfully improved the number of AMF spores by 52.0% over the control under drought conditions (Figure 2).

**FIGURE 3:**
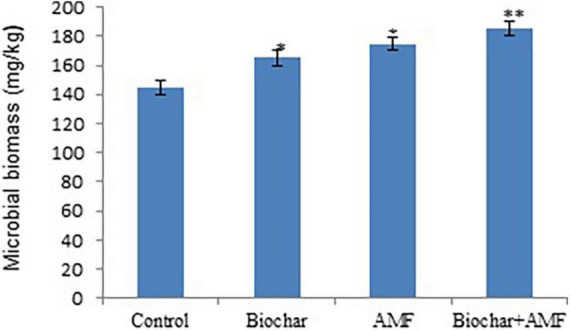
The microbial biomass in soil affected by biochar and AMF under drought conditions. *P = 0.01 and **P = 0.05.

In drought stress, AMF and biochar alone significantly improved the microbial biomass, it increased 14.1 and 20.7%, respectively under drought conditions over the control (Figure 3). In drought stress, dual with biochar and AMF increase microbial biomass by 28.3% in soil compared with the control. The combined application of AMF and biochar under drought stress led to the maximum microbial biomass compared to all treatments.

Under drought stress, a single biochar application significantly enzyme activities of soil (Table 4). The dehydrogenase, alkaline phosphomonoesterase, and FDA activities were enhanced by 23.6, 27.7, and 30.8% when the soil was amended by biochar under water stress. Compared to the control, the application of AMF alone enhanced significantly by 34.0% under water stress. Under water stress, the application of AMF alone, dehydrogenase, and FDA activities increased by 31.5 and 38.1% over the control. In drought conditions, dual applications with AMF and biochar increase soil enzyme activities were greatly better than all treatments. In drought stress, the dual applications of biochar and AMF treatments resulted in 45.9% more alkaline phosphomonoesterase activities than the control. The dual applications with AMF and biochar treatment promote significant dehydrogenase enzyme activity by 46.5% and FDA enzyme activity by 52.2% in soil over the control in drought conditions.

**TABLE 4:** Soil enzymes activities in soil affected by biochar and AMF under drought condition.

Treatments	Alkaline phosphomon- oesterase (μg g^–1^ h^–1^)	Dehydrogenase activity (μg g^–1^ h^–1^)	FDA (fluorescein diacetate) activity (μg g^–1^ h^–1^)
Control	45.67 ± 0.59	38.00 ± 1.01	40.33 ± 0.58
Biochar	58.33 ± 0.58*	47.00 ± 1.00*	52.77 ± 2.08*
AMF	61.20 ± 2.63*	50.00 ± 2.20*	55.73 ± 1.00*
Biochar + AMF	66.67 ± 1.53*	55.67 ± 2.30**	61.40 ± 1.10**

Data are means of three replicates (n = 3). Asterisk differed significantly at *P < 0.05, **P < 0.01, ***P < 0.001.

## Discussion

Drought decreases plant growth parameters and nodule number. Several researchers have reported drought stress reduced plant growth, nodulation, and legume yield (Du et al., 2020; Jabborova et al., 2021e). Rusmana et al. (2020) informed that the growth and yield in *Glycine max* L. Merr. were decreased by water stress.

Numerous studies found that water stress reduced K, P, and N contents in plants (Dimkpa et al., 2017; Hashem et al., 2019) and physiological traits such as relative water content, carotenoids, and chlorophyll contents in legumes (Chowdhury et al., 2016; Kenawy et al., 2022). Ohashi et al. (2006) reported that the photosynthetic pigments in soybean decreased in drought. Geng et al. (2015) and Mariotte et al. (2015) informed water stress declined microbial activity and fungal traits in soil.

Biochar increased plant growth and root morphological traits in soybean under drought in the present study (Tables 1, 2). Similarly, Hashem et al. (2019) and Batool et al. (2015) found that biochar enhanced plant growth parameters in chickpea in water stress. In addition, de Melo Carvalho et al. (2013) reported biochar increased rice biomass in drought conditions. Addition of biochar significantly promotes chlorophyll, N, and C contents in soybean in drought conditions. Several studies show that biochar improved nutrient uptake (Akhtar et al., 2014) and plant physiological traits (Lyu et al., 2016) in various plants under drought stress. The present experiment investigated that biochar improves a significant number of AMF spores, microbial biomass, enzyme activities in soil in a significant number of AMF spores, microbial biomass, and enzyme activities in soil under water stress. Li and Cai (2021) investigated that biochar promoted microbial biomass by 38.0 and 65.9% in water stress. Identical observations recorded by Ahmad et al. (2014) found a helpful impact of biochar on microbial biomass activity.

Numerous studies have demonstrated that inoculation of microorganisms help plants to overcome drought stress and grow well under drought conditions (Kalam et al., 2020; Basu et al., 2021; Hamid et al., 2021; Khan et al., 2021; Sagar et al., 2022). Islam et al. (2022) reported the abundance of mycorrhizae in various host plants and rhizosphere soil. Bastami et al. (2021) reported positive impacts of mycorrhizal fungi and organic fertilizers on quantitative and qualitative traits in *Satureja* species. Researchers have established that AMF improves host plants’ development and water uptake in water stress conditions (Quiroga et al., 2017; Vafa et al., 2021). Identical data were informed by Hashem et al. (2019). Under drought-stressed conditions, AMF improved growth and biomass production, nutrient and water acquisition (Fallah et al., 2021; Jabborova et al., 2021e; Najafi et al., 2021; Saboor et al., 2021). Inoculated with AMF to maize plants significantly increased height by 36.32% and dry weight by 75.73% than the control in water stress (Begum et al., 2019; Rawat et al., 2019).

Arbuscular mycorrhizal fungi inoculation improved plant nutrients in plants under water stress (Baum et al., 2015; Zhao et al., 2015). AMF-inoculated enhanced uptake of minerals “Mg, N, and K” in cucumber in drought conditions (Wang et al., 2008). AMF enhances the significant P content of maize in water stress (Zhao et al., 2015). Similarly, as confirmed by Abdel-Salam et al. (2018), AMF improved damask’s chlorophyll content under water stress. Similarly, Gong et al. (2013) reported that inoculated AMF significantly enhances chlorophyll content and stomatal conductance compared with non-mycorrhizal seedlings in drought. AMF alone considerably promoted the number of AMF spores number, the microbial biomass, and soil enzyme activities in a previous study. Similar findings were reported by Li and Cai (2021) AMF stimulated soil microbial biomass and promoted spores number of AMF in water stress (Hashem et al., 2019).

Combining biochar and AMF under drought stress promoted plant growth and root parameters in plants under drought conditions (Gujre et al., 2021; Mesbah et al., 2021; Ndiate et al., 2021). Budi and Setyaningsih (2013) reported that AMF and biochar promote shoot dry weight in *Melia azedarach* Linn. Harun et al. (2021) informed that combined with AMF and biochar improved “shoot and root biomass” and root length in *Annona muricata* L. Dual applications of AMF and biochar enhance N, C, and chlorophyll content in drought conditions. Similarly, Li and Cai (2021) investigated dual biochar, and AMF improved the P content of maize in water stress. Dual applications with AMF and biochar improve the significant number of AMF spores, microbial biomass, and soil enzyme activities in drought. Hashem et al. (2019) and He et al. (2020) informed that combined application of biochar, and AMF meaningfully stimulated the total number of AMF spores under drought stress conditions. Li and Cai (2021) combined with AMF and biochar greatly improved soil microbial activity and phosphatase activity in soil under water stress.

## Conclusion

Biochar could improve plant growth, root morphological parameters, and soil enzyme activities in water stress conditions. Applications of biochar alone and AMF alone affected soybean tolerance to drought stress. AMF alone promoted plant growth, root morphological traits, chlorophyll content, AMF spores number, and microbial biomass under drought conditions. Dual applications with AMF and biochar showed the best results in drought stress. Combined with AMF and biochar improved plant growth, root morphological traits, and microbial biomass in drought conditions. The dual applications with biochar and AMF can decrease the effects of water stress, helping to improve soybean growth, yield, and soil enzyme activities under drought conditions.

## Data availability statement

The original contributions presented in this study are included in the article/supplementary material, further inquiries can be directed to the corresponding authors.

## Author contributions

DJ: conceptualization, methodology, and writing the original draft. KA, AA, ST, KP, PS, and KV: methodology. DJ, KA, ON, MA, and RS: formal analysis and writing—review and editing. PP: conceptualization, formal analysis, open access fund acquisition, and writing—review and editing. ON: writing—review and editing and fund acquisition. All authors contributed to the article and approved the submitted version.

## Conflict of interest

The authors declare that the research was conducted in the absence of any commercial or financial relationships that could be construed as a potential conflict of interest.

## Publisher’s note

All claims expressed in this article are solely those of the authors and do not necessarily represent those of their affiliated organizations, or those of the publisher, the editors and the reviewers. Any product that may be evaluated in this article, or claim that may be made by its manufacturer, is not guaranteed or endorsed by the publisher.
